# Long-term effects of adolescent stress on neophobic behaviors in zebra finches are modulated by social context when in adulthood

**DOI:** 10.1016/j.yhbeh.2017.02.004

**Published:** 2017-04

**Authors:** Michael G. Emmerson, Karen A. Spencer

**Affiliations:** University of St Andrews, School of Psychology & Neuroscience, St Mary's Quad, South Street, St Andrews, Fife KY16 9JP, Scotland, United Kingdom

**Keywords:** Corticosterone, Developmental stress, Adolescence, Novel environment, Social buffering, HPA axis, Programming, Plasticity

## Abstract

Experiencing stress during adolescence can increase neophobic behaviors in adulthood, but most tests have been conducted in the absence of conspecifics. Conspecifics can modulate responses to stressors, for example by acting as ‘social buffers’ to attenuate the aversive appraisal of stressors. Here, we investigate the long-term effects of adolescent stress on the behavioral responses to novel stimuli (a mild stressor) across social contexts in an affiliative passerine bird, the zebra finch. During early (days 40–60) or late (days 65–85) adolescence the birds (n = 66) were dosed with either saline or the hormone corticosterone (CORT). CORT was given in order to mimic a physiological stress response and saline was given as a control. In adulthood, the birds' behavioral responses to a novel environment were recorded in both the presence and absence of conspecifics. An acute CORT response was also quantified in adolescence and adulthood. Our findings show clear evidence of social context mediating any long-term effects of adolescent stress. In the presence of familiar conspecifics no treatment effects were detected. Individually, birds dosed with CORT in early adolescence were slower to enter a novel environment, spent more time perching in the same novel environment, and, if female, engaged in more risk assessment. Birds dosed in late adolescence were unaffected. No treatment effects were detected on CORT, but adolescents had a higher CORT concentration than adults. Our results are the first to suggest that familiar conspecifics in adulthood can buffer the long-term effects of stress that occurred during early adolescence.

## Highlights

• Elevated corticosterone in adolescence increases neophobic behaviors in adulthood.
• Familiar conspecifics in adulthood buffer long-term effects of adolescent stress.
• Corticosterone may be an endocrine mechanism behind sustained changes in behavior.

## 1 Introduction

Stimuli that are perceived as a (potential) threat activate the hypothalamic-pituitary-adrenal axis, a neuroendocrine cascade regulating the secretion of glucocorticoids (GCs), such as corticosterone (CORT) in rodents and birds (Romero, 2004, Sapolsky et al., 2000). GCs alter many physiological systems in order to facilitate an acute ‘stress response’ (McEwen and Wingfield, 2010, Sapolsky et al., 2000). The behavioral consequences of a stress response include increased locomotor activity, risk assessment, and risk avoidance (Haller et al., 1998, McNaughton and Corr, 2004, Rodgers et al., 1999), but increased risk-taking has also been noted (Martins et al., 2007, Toledo-Rodriguez and Sandi, 2011). Exposure to stressors during early post-natal development can have sustained effects on an adult's stress response (Sachser et al., 2011, Lupien et al., 2009, Monaghan, 2008), with adult neophobic behaviors and stressor-induced CORT concentration higher in those animals exposed to developmental stress compared to control animals (Hollis et al., 2013). GCs appear to be the endocrine mechanism behind the long-term effects of developmental stress, as direct elevation of CORT during development induces long-term effects on both neophobic behavior and stressor-induced CORT secretion in a number of bird species (Schoech et al., 2012), including zebra finches (*Taeniopygia guttata*; Spencer and Verhulst, 2007, Spencer et al., 2009). CORT likely has sustained effects on adult stress responses by altering neural expression of GC receptors, subsequently heightening the concentration of CORT secreted in response to stressors and in turn heightening the expression of neophobic behavior (Banerjee et al., 2012, Isgor et al., 2004, Zimmer and Spencer, 2014).

Adult social interactions have influence over responses to stressors, with conspecific presence attenuating or ‘buffering’ aversive responses to novelty and other stressors in several species of rodents (Beery and Kaufer, 2015, DeVries et al., 2003, Hennessey et al., 2009, Kikusui et al., 2006) and primates (Gunnar and Hostinar, 2015, Sanchez et al., 2015). In birds, familiar conspecifics (vs. single housing) also appear to act as social buffers noted by reduced latencies to feed from a feeder in the presence of a novel object in zebra finches (Coleman and Mellgren, 1994) and budgerigars (*Melopsittacus undulatus*; Soma and Hasegawa, 2004). However, European starlings (*Sturnus vulgaris*) do not appear to act as social buffers to one another (Apfelbeck and Raess, 2008). Absence of a social buffering effect in starlings likely reflects a floor effect, as so few birds engaged in the task of foraging from a familiar feeder after a novel object was attached to the feeder (Apfelbeck and Raess, 2008). Familiar conspecifics therefore tend to attenuate neophobic behaviors in adulthood, but no work has investigated whether familiar conspecifics can attenuate neophobic behaviors that were heightened in response to developmental stress.

Adolescence is a developmental stage that begins with puberty and ends with sexual maturity (Brown and Spencer, 2013, Spear, 2000). During adolescence, animals leave the natal home and become exposed to stressors, such as unfamiliar environments and predators (Spear, 2000, Yoder et al., 2004). Stressor exposure during adolescence can have long-term effects on an adult's stress response (Eiland and Romeo, 2013). Animals exposed to stressors during adolescence, compared to controls, display more neophobic behaviors in adulthood (Hollis et al., 2013) and have a higher CORT concentration in response to a stressor in adulthood (Isgor et al., 2004, Pohl et al., 2007). The neural development of HPA axis regulators, such as the amygdala, continues to occur during adolescence; with more pronounced changes occurring earlier in adolescence (Andersen and Teicher, 2008). Adolescents are also sensitive to steroid hormones, with neural sensitivity to steroid hormones declining with increasing age (Schulz et al., 2009). Resultantly, exposure to stressors during adolescence appears to have more pronounced effects on later-life responses to stressors the earlier in adolescence the developmental stress occurs (Tsoory and Richter-Levin, 2006). However, little work has attempted to investigate the importance of age-related changes in hormone sensitivity on the formation of adult phenotypes. In addition, the long-term effects of adolescent stress have only been quantified in an individual context. Whether familiar conspecifics in adulthood can buffer the long-term effects of adolescent stress on adult neophobic behavior and the stressor-induced secretion of CORT remain to be tested.

In the current study we investigated the long-term effects of adolescent stress on adult responses to novel stimuli, both individually and in the presence of familiar conspecifics, and the CORT response to a capture/restraint stressor. We used a highly social passerine bird, the zebra finch, expected to be sensitive to changes in social context. Zebra finches begin puberty around postnatal day 30 and reach sexual maturity by postnatal day 100 (Zann, 1996). Birds were orally dosed with CORT during adolescence in order to mimic a physiological response to a stressor. Birds were dosed either in early adolescence (postnatal days 40–60) or in late adolescence (postnatal days 65–85). Two treatment periods were chosen in order to investigate whether the effects of adolescent stress decline with age. The specific ages were chosen as they are ten days either side of behavioral changes that occur during adolescence in zebra finches. The birds begin to spend more with unfamiliar conspecifics by postnatal day 50 (Adkins-Regan and Leung, 2006) and begin to display sexual behaviors by postnatal day 75 (Zann, 1996). In adulthood, behavioral responses to a novel environment (avoidance, risk assessment, locomotor activity) were quantified after the birds had been single housed (individual context) and when the birds were in their home cages with their familiar cage mates (group context). CORT secretion in response to a capture/restraint stressor was also quantified, first in adolescence shortly after the treatment period ended and then in adulthood after behavioral testing was completed. We predicted that (1) bird's treated with CORT in adolescence would display more avoidant, locomotor, and risk assessment behaviors in an individual context novel environment in adulthood compared to saline treated birds, (2) no effects of adolescent CORT would be detected in a group context novel environment, (3) the secretion of CORT in response to capture/restraint would be higher in birds treated with CORT during adolescence compared to saline treated birds, and (4) birds treated with CORT in early adolescence would display more avoidant, locomotor, and risk assessment behaviors and have a higher CORT response than birds treated with CORT in late adolescence.

## 2 Methods

### 2.1 Experimental design

All birds were housed in a single room with a 12:12 light-dark cycle (lights on at 07:00), temperature at 22 ± 2°, and relative humidity at 55 ± 5%. All appropriate ethical guidelines and requirements were adhered to, as set out in the Principles of Laboratory Animal Care (NIH, Publication No. 85–23, revised 1985) and the UK Home Office Animals (Scientific Procedures) Act 1986 under project licence 70/8159 and personal licences IDFA58352, IEBE43CFF, and 60/13261.

Adult zebra finches (n = 20 male, 20 female) from an in-house breeding stock were housed in one of two 100 cm long × 100 cm high × 50 cm deep white wrought iron cages (10M, 10F per cage) and observed for two one hour sessions per day (one 8:30–13:00, one 13:00–17:30) for 14 days. Birds had ad libitum access to seed (Food for Finches, Johnson & Jeff, UK) hoppers, water hoppers, and water baths. Spinach was provided on days one, six, and eleven. When two birds engaged in any mating behavior (following, directed song, mounting) on three consecutive sessions the pair were removed from the cage and housed together. Each pair (n = 20) was housed in one half of a breeding cage (MB 3612 Metal Double Breeding Cage, R.J. Leigh Ltd., UK) with each cage half measuring 60 cm × 50 cm × 50 cm (length × height × depth). Each cage half was separated by a solid metal divider. The cage bases were covered with wood pellets (Stovies Wood Pellets, Arbuthnott Wood Pellets Ltd., UK) and contained two plastic perches. Birds had ad libitum access to seed and water hoppers, a grit tray, and a water bath like that for the partner choice cages.

A cardboard nest box was attached to the outside of the cage with an entrance facing the cage interior. Hay and jute fibre nesting materials were given daily until the beginning of incubation. Nests were inspected daily. Egg food (CéDé Premium Egg Food, Belgium) was provided daily until chicks reached nutritional independence. Hatch order can have long-term effects on adult exploratory behavior (Mainwaring and Hartley, 2013), so an attempt was made to synchronise hatching. Eggs were removed on the day they were laid and replaced with a fake ‘Fimo’ egg (Staedtler Fimo Soft Oven Hardened Modelling Clay (white), UK). After two consecutive days of not laying, eggs were returned to the nest to allow incubation. Eggs were candled at day seven, with any infertile clutches removed to allow relaying. On post-hatch day (PHD) 5, the chicks were given a temporary ID (coloured nail polish applied to each leg, re-applied on PHD 8) before being given a permanent ID (ne uniquely numbered orange leg ring and one coloured leg ring (pink, yellow, light blue, or white) on PHD 10. A blood sample (approximately 5 μl) was taken from the brachial vein of all chicks on one day between PHD 12–15 for molecular sexing (see below). After nutritional independence was achieved (day 35), the offspring (n = 66) were separated from their parents and re-housed in same-sex, non-sibling triplets in cages identical to the home cage (i.e. half of a full breeding cage with a solid divider separating the two halves and therefore two triplets). Triplets were matched for age (± 2 days) and mass (within 1 g). The birds remained in these triplets until the end of the experiment. Each triplet was randomly distributed across four treatments: early adolescent CORT (E-CORT; n = 9M, 9F), early adolescent saline controls (E-SAL; n = 9M, 6F), late adolescent CORT (L-CORT; n = 9M, 9F), and late adolescent saline controls (L-SAL; n = 9M, 6F).

### 2.2 Molecular sexing

DNA was extracted using a DNeasy Blood and Tissue kit (Qiagen Ltd.) following the protocol for nucleated erythrocytes. Extracted DNA was used to perform PCR to amplify CHD gene fragments with the primer pair P2 (5′-TCTGCATCGCTAAATCCTTT-3′)/P17 (5′-GAAGAAAATAATTCCAGAAGTCCA-3′) that has previously been developed for sexing zebra finches (Arnold et al., 2003). All PCR reactions were run in a final volume of 10 μl consisting of 0.8 μl of P2 and P17 primers, 200 μM of each dNTP, 0.8 μl of 25 mM MgCl_2_, 2 μl (5 ×) of GoTaq Flexi buffer (Promega, UK), 0.35 units of GoTaq polymerase (Promega, UK), and 100 μM of target DNA. All reactions were carried out on a TGradient 96 Biometra thermal cycler (Biometra GmBH, Goettingen, Germany) at: 94 °C for 2 min, 29 cycles of 94 °C for 30 s, 49 °C for 45 s, 72 °C for 40 s, 49 °C for 1 min, and 72 °C for 5 min. PCR products were separated by electrophoresis on 2% agarose gels stained with ethidium bromide and visualized using a Bio-Rad Gel Doc XR + system (Bio-Rad Laboratories Ltd.).

### 2.3 Pilot study

In order to determine the dose for each treatment group a pilot study was undertaken. Early adolescent birds (n = 5M, 5F) aged 40–60 days (mean = 51.45) and late adolescent birds (n = 5M, 5F) aged 65–85 days (mean = 73.14) were housed in same-sex age-similar cages of either two or three birds. Birds were captured between 9 and 11 am and a blood sample (approximately 40 μl) was collected within three minutes of disturbance to ensure an accurate baseline sample was collected (Romero, 2004). Each bird was then restrained in a black cloth bag for 10 min, after which a second sample (approximately 30 μl) was taken. Samples were put on ice, centrifuged at 3500 rpm for 10 min, and the plasma was then extracted before being stored at − 20 °C. A radioimmunoassay was performed to determine the CORT concentration in each plasma sample (Spencer et al., 2009). Full assay details can be found in section *2.5.4* Radioimmunoassay of this paper. All samples were run in a single assay. Intra-assay co-efficient of variation was 10.72%. The CORT concentration at 15 min was 12.14 ng/ml (SD = 6.34) irrespective of age (see supplementary data for data analysis). Previous CORT dosing studies have administered CORT at a concentration two standard deviations above the mean concentration of stressor-induced CORT in order to mimic a stress response that is within a physiologically relevant range (Spencer et al., 2009). We therefore aimed to elevate circulating CORT to 25 ng/ml (2 standard deviations above 12.14 ng/ml) in both the groups that were treated with CORT.

### 2.4 Experimental manipulation

To raise the circulating plasma concentration of CORT to 25 ng/ml, the CORT dosed birds were treated with a scaled dose of CORT based on Spencer et al. (2009). The CORT dosed birds were administered 10 μl of 0.36 mg/ml corticosterone (Sigma Aldrich, UK) dissolved in autoclaved saline (PBS, Sigma Aldrich, UK; 0.01 M, pH 7.4) twice per day; total daily CORT of 7.2 μg. The control birds were fed 10 μl of autoclaved saline twice per day. The birds were treated with the CORT or saline doses every day between PHD 40–60 (E-CORT, E-SAL) or PHD 65–85 (L-CORT, L-SAL). Birds were fed cucumber cubes (approximately 0.5 cm^3^) containing the CORT or saline dose via a plastic mesh box that was attached to the front of the home cage, designed so that only one bird had access to the cucumber at any one time. When a bird voluntarily entered the box the experimenter placed an individual cube of cucumber through one of the holes in the box and onto a dish at the back of the box. If a dosed bird re-entered the box no cucumber was given. Each dosing box was attached to the cage until all birds had consumed the appropriate dose, which lasted no more than 10 min. To habituate the birds to the use of this procedure all parental birds were trained to consume cucumber from the dosing box for 15 min per day starting when the offspring were PHD 5. All fledglings were observed following the parents into the dosing box by PHD 19–23 and independently entering the box and consuming cucumber by age 28–33 days.

### 2.5 Behavioral measures

#### 2.5.1 Adolescent approach/avoidance behavior

During the treatment period a timer was started when the dosing box was attached to the home cage. The latency (seconds) for each bird to enter the dosing box was recorded to determine whether the CORT treatment was affecting the birds within adolescence. A bird was defined as being in the dosing box when both feet were fully on the floor of the dosing box.

#### 2.5.2 Adult novel environment tasks

In adulthood (PHD 116–139) behavioral responses to a mild stressor, a novel environment, were quantified. Birds were tested twice: once alone and once in a familiar social group, with test order counterbalanced across treatments and sexes. The novel environments were white powder coated steel cages measuring 120 cm × 50 cm × 50 cm (length × height × depth). The cages were split down the middle with a solid divider to create two separate compartments measuring 60 cm × 50 cm × 50 cm (length × height × depth). The cages were identical to those in the home room, with each separate compartment identical to the home cage. In one of the separate environments, novel objects were attached to the perches. In the individual context the objects were a pink ball, a pyramid of three coloured blocks, and two intertwined dark blue pipe cleaner rings. In the group context the objects were a yellow tub, a green pipe cleaner helix, and a ‘U’ of coloured blocks. Each object was baited with a shredded spinach leaf as a food reward. The novel environments were selected after a pilot study of 12 adult non-experimental birds (6M, 6F) determined them to be sufficiently and similarly aversive in an individual context (latency (minutes) to enter novel compartment, individual context objects, M = 21.09, SD = 5.481; group context objects, M = 22.32, SD = 4.42). The compartment that contained the novel objects was considered a novel environment, whereas the compartment that did not contain novel objects was considered familiar as it was identical to the home cage. In the individual context, birds were captured and placed by themselves in the familiar compartment of a test cage that was in a room separate from the holding room. All of the birds from a triplet were tested at the same time whilst in acoustic, but not visual, contact to one another. Testing began twenty four hours after individual housing. In the group context, birds were briefly captured and then immediately returned to the home cage 24 h prior to testing to control for the handling effects that were present in the individual context. As previously described (see 2.1. Experimental design), the home room housing had triplets housed in opposing halves of full length cages. In the group context, birds were therefore tested whilst birds in the opposing cage compartment were undergoing individual context testing. After removing birds for the individual context testing the home cage compartment was cleaned and novel objects were then placed in the cage to create the group context novel environment.

During testing, behavior was quantified during a 90 min session consisting of two phases: pre-exposure (30 min) and exposure (60 min). During pre-exposure the measures of behavior recorded were the *number of hops between perches* and the *number of head turns.* Head turns were recorded as a measure of risk assessment. Due to the lateral placement of the eyes birds scan their surroundings to acquire more information (Fernandez-Juricic, 2012), perhaps to assess risk as has been noted in chaffinches (*Fringilla coelebs*; Jones et al., 2007). Head turns were defined as ninety degree turns of the head, with zero degrees being head facing forward and beak aligned with the midline of the body. After pre-exposure, a cage divider was removed to reveal the novel compartment. During exposure the measures of behavior were *latency to enter novel compartment*, *duration of time perching in the novel compartment*, *number of hops between perches*, and *number of ninety degree head turns*. Novel compartment side (left or right) was counter-balanced across treatment groups and sexes.

#### 2.5.3 Physiological response to stress

The acute CORT response to a standardised capture/restraint stressor (Spencer et al., 2009) was conducted in adolescence after each treatment period had ended (early dosed, PHD 63 ± 2; late dosed, PHD 88 ± 2) and then in adulthood (PHD 130–145). The brachial vein was pricked with a 27-gauge needle tip and a blood sample (40 μl) was collected into a heparinised capillary tube within three minutes after entering the holding room to ensure an accurate basal sample (Romero, 2004). Birds were then placed in individual black cloth bags and further samples (30 μl) were taken at 10 min and 30 min after entering the holding room. Samples were placed on ice until they could be centrifuged and the plasma extracted. Plasma was stored at − 20 °C until radioimmunoassay.

#### 2.5.4 Radioimmunoassay

10–30 μl samples of plasma were extracted with 1 ml of diethyl ether. CORT concentration was then determined via radioimmunoassay (Spencer et al., 2009) with the use of anti-CORT antiserum (Esoterix Endocrinology, USA B3–163) and [1,2,6,7-3*H*]-CORT label (Perkin Elmer, UK). The detection limit was 0.05 ng/ml and extraction efficiency ranged between 71.24 and 100%. All samples were run in duplicate across six assays, with all samples from a single individual run in the same assay with groups and sexes distributed across assays. 50% binding (ng/ml) for each assay was 0.65, 0.72, 0.68, 0.64, 0.72, and 0.64. Intra-assay coefficients of variation (%) for each assay were 11.11, 12.42, 9.3, 8.13, 10.07, and 11.34. Inter-assay coefficient of variation (%) was 14.64.

#### 2.5.5 Data analysis

Statistical analysis was conducted using SPSS version 22. All data were analysed using linear mixed models (LMM) with brood size entered as a random factor. Treatment, sex, and the interaction between the two were entered as fixed factors in all models. Home cage was entered as a random factor in group context models to account for the influence of cage partners. Residuals of all dependent variables were checked for normality (Shapiro-Wilk, p > 0.05). In some instances, data were square root or log10 transformed to achieve normally distributed residuals. Data transformations, when applicable, are reported in brackets after each behavioral variable alongside the social context in which the transformation applies.

For adolescent latency to enter the dosing box, twenty trial days were reduced to five trials per bird by calculating the mean latency for days 1–4, 5–8, 9–12, 13–16, and 17–20. In a single model, latency (seconds) to enter dosing box (square root) was entered as a dependent variable with trial number (1–5) entered as a fixed and repeated measure. For adult behavior, eight separate models were used with analyses performed separately for each behavior and context. Latency (seconds) to enter novel environment (individual = square root), duration of time (seconds) perching in novel environment (individual and group = square root), number of hops (individual and group = square root), and number of head turns (group = square root) were entered as dependent variables in the models. Session (pre-exposure, exposure) was entered as a fixed and repeated measure in hop and head turn models. A second person re-coded adult behavior for eight birds (one male, one female per group) to determine inter-observer reliability. Intra-class correlation coefficients revealed high agreement for all measures (r ≥ 0.918). For hormone data, a single model was used with log10 transformed CORT concentration (ng/ml) entered as a dependent variable with duration of restraint (0, 10, and 30 min) and age (adolescent, adult) entered as fixed factors and repeated measures. To investigate the relationship between behavior and hormones Spearman's correlation models were used with separate models conducted for each behavioral test context. An alpha level of 0.05 was used as the threshold for statistical significance in all models. Significant main effects and interactions were further investigated with Bonferroni post hoc tests. Cohen's d was calculated as a measure of effect size for post hoc comparisons. All data presented are means ± standard error of the mean.

## 3 Results

Output tables for each model can be found in the supplementary data.

### 3.1 Adolescent approach/avoidant behavior

**Fig. 1:**
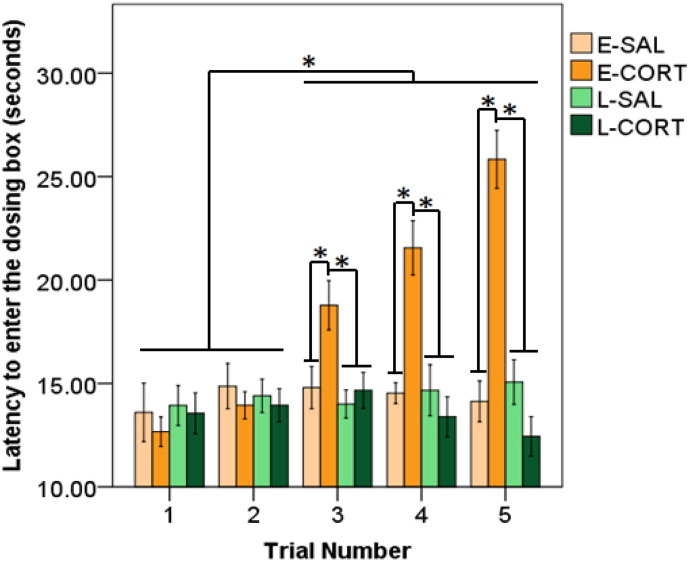
Latency to enter dosing box (seconds) during adolescent dosing split by early and late adolescent saline treatment (E-SAL, L-SAL) and corticosterone treatment (E-CORT, L-CORT). Birds took longer to enter the dosing box during the latter three trial blocks, but the effect was driven by the E-CORT birds. An * denotes significant differences (p < 0.05).

Adolescent treatment had a significant effect on the latency to enter the dosing box (F_3,58_ = 7.37, p ≤ 0.001; Fig. 1), with E-CORT birds taking longer to enter the dosing box in comparison to all other groups (p's ≤ 0.027; d's ≥ 0.741). These effects were dependent on the trial number (F_12,58_ = 16.09, p ≤ 0.001), with E-CORT birds taking longer to enter the dosing box than all other groups between trial days 9–12, 13–16, and 17–20 (p's ≤ 0.035; d's ≥ 0.864). All other effects were not significant (p's ≥ 0.143).

### 3.2 Adult neophobic behavior

**Fig. 2:**
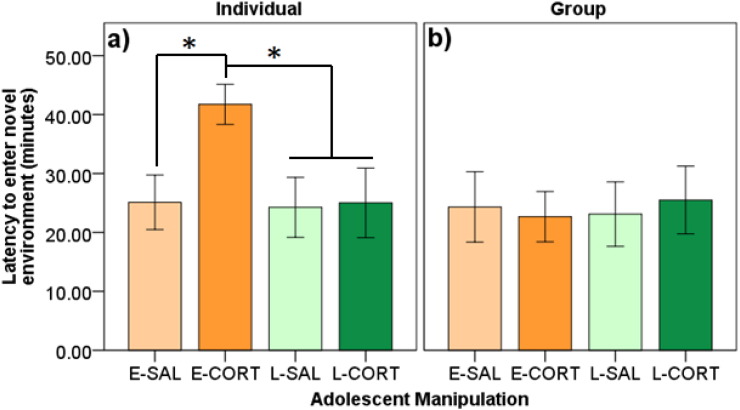
Latency (minutes) to enter the novel environment in A) individual and B) group novel environment tasks split by early and late adolescent saline treatment (E-SAL, L-SAL) and corticosterone treatment (E-CORT, L-CORT). E-CORT birds took longer to enter the novel environment compared to all other groups when tested individually, but no effects were found when tested in groups. An * denotes significant differences (p < 0.05).

Latency to enter the novel environment was affected by CORT in the individual context (F_3,58_ = 7.66, p ≤ 0.001; Fig. 2a), but not the group context (F_3,14.30_ = 0.03, p = 0.994; Fig. 2b). Individually, E-CORT birds entered the novel environment later than all other groups (p's ≤ 0.016; d's ≥ 0.88). No other effects were detected in either context (p's ≥ 0.139).

**Fig. 3:**
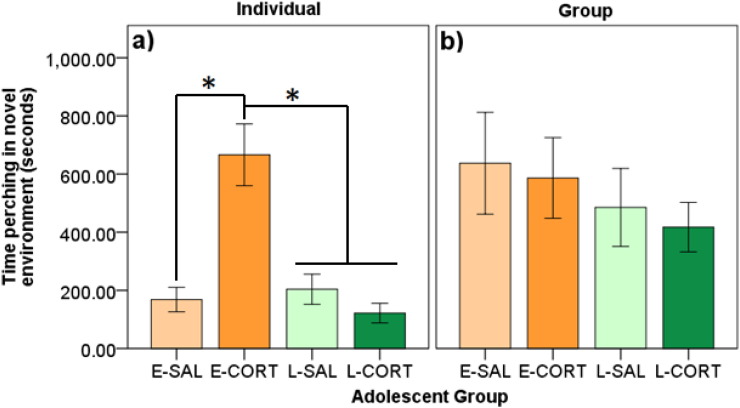
Duration of time (seconds) perching in the novel environment in A) individual and B) group novel environment tasks split by early and late adolescent saline treatment (E-SAL, L-SAL) and corticosterone treatment (E-CORT, L-CORT). E-CORT birds spent more time perching in the novel environment compared to all other groups when tested individually, but no effects were found when tested in groups. An * denotes significant differences (p < 0.05).

Duration of time perching in the novel environment was affected by adolescent treatment in the individual context (F_3,56.66_ = 13.49, p ≤ 0.001; Fig. 3a), but not the group context (F_3,14.19_ = 0.203, p = 0.893; Fig. 3b). Individually, E-CORT birds perched for a longer duration in the novel environment compared to other groups (p's < 0.001; d's ≥ 1.41). No other effects were detected in either context (p's ≥ 0.148).

**Fig. 4:**
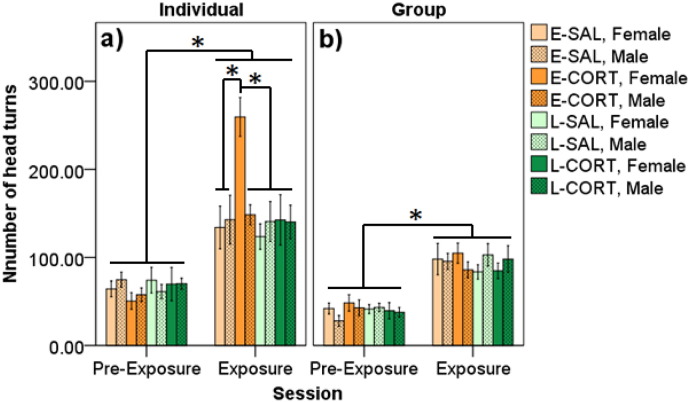
Number of head turns during pre-exposure and exposure sessions in A) individual and B) group novel environment tasks. Data are split by early and late adolescent saline treatment (E-SAL, L-SAL) and corticosterone treatment (E-CORT, L-CORT) for each sex. Birds engaged in more head turns during exposure compared to pre-exposure. In exposure, E-CORT females engaged in more head turns compared to all other group and sex combinations when tested individually. However, no differences were found when tested in groups. An * denotes significant differences (p < 0.05).

Number of head turns was greater during the sixty minute test relative to the thirty minute pre-test session in both contexts (F_1,58_ ≤ 128.52, p's < 0.001). Treatment interacted with pre- vs. post- exposure session in the individual context (F_3,58_ = 6.25, p = 0.001; Fig. 4a), but not the group context (F_3,58_ = 0.40, p = 0.752; Fig. 4b). Individually, E-CORT birds engaged in more head turns when exposed to the novel environment in contrast to other groups (p's ≤ 0.015; d's ≥ 0.85). Sex mediated an interaction between CORT and session in an individual context (F_3,58_ = 4.21, p = 0.009), but not the group context (F_3,58_ = 0.47, p = 0.706). Individually, E-CORT females engaged in more head turns when exposed to a novel environment in contrast to all other sex and group combinations (p's ≤ 0.001; d's ≥ 1.55; Fig. 4a). All other effects were not significant (p's ≥ 0.075).

**Fig. 5:**
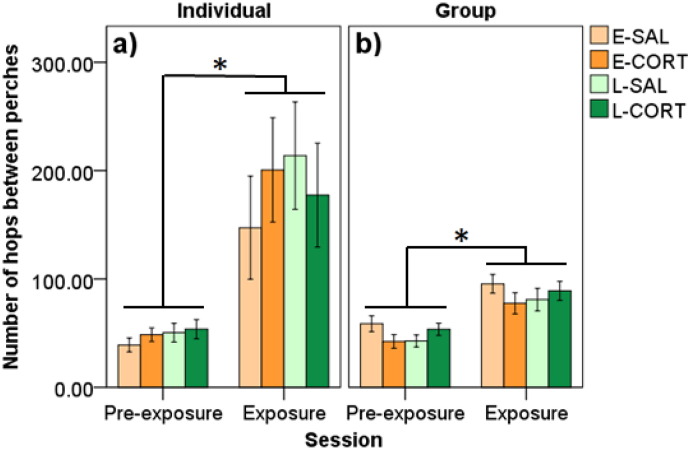
Number of hops during pre-exposure and exposure sessions when tested in A) individual and B) group novel environment tasks. Data are split by early and late adolescent saline treatment (E-SAL, L-SAL) and corticosterone treatment (E-CORT, L-CORT). Birds hopped more during exposure compared to pre-exposure, but no group and/or sex effects were detected. An * denotes significant differences (p < 0.05).

Number of hops was greater during the sixty minute test session than during the thirty minute pre-test session irrespective of adolescent treatment in both contexts (individual = F_1,58.12_ = 20.26, p ≤ 0.001; group = F_1,58.74_ = 70.46, p ≤ 0.001; Fig. 5a and b, respectively). All other effects were not significant (p's ≥ 0.316).

### 3.3 Physiological stress response

**Fig. 6:**
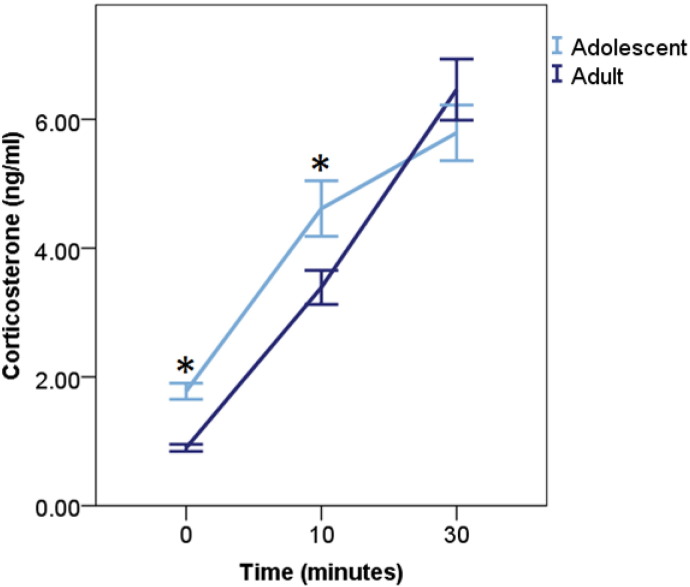
Corticosterone concentration (ng/ml) secreted during a capture and restraint stressor averaged across the groups and sexes and then split by age. Corticosterone concentration was higher in adolescents than adults at baseline and 10 min into restraint, but no age differences were found at 30 min into restraint. An * denotes a significant difference between ages within a specific sampling time point (p < 0.05).

Averaging across all treatments the CORT concentration in response to capture/restraint was influenced by restraint duration (F_2,58.03_ = 220.36, p < 0.001), with more CORT detected at 10 min and 30 min vs. baseline samples, and more CORT detected at 30 min vs. 10 min (p's < 0.001; d's > 0.70). CORT secretion was also influenced by age, with a higher concentration of CORT secreted during adolescent sampling than adult sampling across treatments (F_1,57.94_ = 16.38, p ≤ 0.001). The effect of age was dependent on sampling time (F_2,57.93_ = 20.72, p ≤ 0.001; Fig. 6), as across all treatments adolescents secreted a higher concentration of CORT than adults at baseline and 10 min samples (p's ≤ 0.040; d's ≤ 0.43), but not 30 min (p = 0.244; d = 0.14). All other effects were not significant (p's ≥ 0.094).

### 3.4 Correlations

In the individual context, number of hops during exposure to a novel environment was positively correlated with number of head turns during exposure (r = 0.345, p = 0.005). In addition, number of hops during pre-exposure was inversely correlated with adolescent CORT concentration 10 min into restraint (r = − 0.256, p = 0.038) and the number of head turns during pre-exposure was inversely correlated with adult basal CORT (r = − 0.299, p = 0.015). All other effects were not significant (p's ≥ 0.124).

In the group context, latency to enter the novel environment was inversely correlated with duration of time spent perching in the novel environment (r = − 0.630, p < 0.001) and positively correlated with number of head turns during exposure (r = 0.247, p = 0.045). In addition, number of hops during pre-exposure was positively correlated with number of hops during exposure (r = 0.439, p < 0.001) and inversely correlated with number of head turns during pre-exposure (r = − 0.281, p = 0.022). All other effects were not significant (p's ≥ 0.116).

## 4 Discussion

Our results clearly show that early adolescent stress can have long-term effects on behavioral responses to novelty in adulthood. In an individual context E-CORT birds showed an initially more neophobic response by taking longer to enter the novel environment, but also appeared more risk-taking as birds went on to spend more time perching in the novel environment. E-CORT females also appeared somewhat more neophobic than other birds, noted by a greater increase in novelty-induced head turns. Our findings therefore corroborate and extend upon those of Spencer and Verhulst (2007), suggesting exposure to CORT during the first sixty days of postnatal life can have a sustained influence on adult behavioral responses to novelty in the zebra finch. However, the two studies indicate that pre-pubertal and adolescent stress can have independent long-term effects on adult responses to novelty. Behavioral measures in the individual context were largely not correlated, possibly indicating that developmental stress has multiple independent effects on behavioral responses to novel stimuli rather than inducing a single integrated risk-avoidant phenotype.

Any effects of adolescent stress on behavioral responses to novelty were dependent upon social context. Adolescent CORT had no effect on adult novelty responses when birds were tested with familiar conspecifics. Previous work has shown that adult zebra finches can act as social buffers to one another (Coleman and Mellgren, 1994). Our results suggest that the long-term effects of early adolescent stress can be buffered by familiar conspecifics when in adulthood. An alternative explanation is that adolescent CORT may exaggerate the stress of handling and separation, thereby creating greater pre-test stress in an individual context that exaggerates behavioral responses to novelty. However, twenty four hours prior to testing both individual and group context birds were briefly captured to control for handling stress effects. Zebra finches have elevated basal CORT twenty-four hours after separation from a cage mate and subsequent single housing (Remage-Healey et al., 2003), suggesting separation can be a stressor. However, no group differences were detected on pre-test behaviors (locomotor activity and risk assessment) in this experiment. The most parsimonious explanation for the reported effects is that familiar conspecifics in zebra finches act as social buffers to potential adversity, as has been noted previously in other social species including birds (Coleman and Mellgren, 1994, Soma and Hasegawa, 2004), rodents (Beery and Kaufer, 2015, Hennessey et al., 2009), and primates (Gunnar and Hostinar, 2015, Sanchez et al., 2015). However, we are the first to show that long-term effects of adolescent stress are buffered by familiar conspecifics in adult birds. In the group context the latency to enter the novel compartment was inversely related to the time spent in the novel compartment and positively related to number of head turns when exposed to the novel compartment, suggesting that birds in a group context have an integrated neophobic response to novelty characterised by higher levels of risk-avoidance and risk assessment. The correlation between latency and head turn measures also supports our suggestion that ninety degree head turns in the zebra finch are a response to novelty like that seen in chaffinches and plausibly reflect assessment of risk (Jones et al., 2007).

Neophobic behavioral responses typically consist of risk-avoidance (Haller et al., 1998, McNaughton and Corr, 2004), but evidence of greater risk-taking has also been reported (Martins et al., 2007, Toledo-Rodriguez and Sandi, 2011). E-CORT birds in an individual context appeared more risk-avoiding (longer to enter novel environment) then more risk-taking (more time perching in novel compartment). One possible explanation for the contrary effects is that the longer duration of time spent in the novel compartment does reflect risk-avoidance, with E-CORT birds engaging in more stress-induced freezing (Hagenaars et al., 2014, Koolhaas et al., 1999). However, freezing would be expected to lower measures of activity and in the current study CORT dosing did not affect locomotor activity. E-CORT birds, consequently, may have actually become more risk-taking as the task progressed. The effect appears sex-dependent, as E-CORT females engaged in more risk assessment indicted by a greater increase in head turns in response to a novel environment. Head turns are a novel measure of risk assessment in zebra finches, so alternative explanations are available. Birds may engage in more head turns as an attempt to locate another bird due to greater separation stress. However, this did not seem likely given the correlation between head turns and latency to enter the novel compartment in the group context. In addition, increased head turns in E-CORT birds across the entire test session would be predicted if more separation stress was occurring. Instead, E-CORT females specifically increased head turn number during exposure to a novel environment. Head turns appear to be a response to novelty, plausibly to assess risk. Number of head turns, like number of hops, was higher during exposure compared to pre-exposure; an unsurprising effect given that the exposure session was twice as long as pre-exposure. However, adult locomotor activity was not affected by developmental exposure to CORT; an effect that has been reported previously (Spencer and Verhulst, 2007). CORT may not have long-term effects on activity, but short-term effects have not been studied. Lower locomotor activity during adolescent dosing could explain why E-CORT birds took longer to enter the dosing box in adolescence. Developmental stress did not influence activity, but a group context appeared to lower the increase in activity compared to an individual context. We cannot confirm this due to modelling differences between contexts, so future work may wish to investigate how social groups modulate a bird's activity levels in response to novelty.

Sustained effects of developmental experiences, such as stress, are known to be more pronounced the earlier in development the experiences occur (Andersen and Teicher, 2008, Tsoory and Rrichter-Levin, 2006). As predicted, early adolescents were more responsive to CORT than late adolescents. Early adolescents could be more sensitive than late adolescents to steroid hormone action (Schulz et al., 2009). However, age-related differences in CORT secretion are another explanation. For example, adolescent rats, compared to adults, secrete more CORT during an acute stress response to a restraint stressor (Gomez et al., 2002, Ver Hoeve et al., 2013), perhaps permitting more sustained effects of adolescent stress (Eiland and Romeo, 2013). In zebra finches, a previous study investigating within-adolescent changes in an acute CORT response did not compare across ages (Crino et al., 2014). However, here we report that both early and late adolescent zebra finches secrete more CORT at baseline and 10 min after a restraint stressor compared to adults. As no differences exist within adolescence (i.e. early vs. late), age-related differences in CORT secretion do not appear to mediate the effects of adolescent stress. However, we expected birds to begin reducing CORT secretion within 30 min as was observed in Spencer et al. (2009) but this did not materialise; a similar effect was noted in Crino et al. (2014). Time of decline has previously been shown to be the sole measure of the dynamic CORT response to be affected by developmental stress (Welberg and Seckl, 2001, Zimmer et al., 2013) and may have been affected here but remained unmeasured. Future work should therefore sample birds at a later time point (s). In the current study, adolescent zebra finches had higher CORT concentration than adults at both basal and 10 min time points. One explanation is order effects, as adolescent birds were exposed to the restraint stressor before adults. Birds may have habituated to the restraint stressor resulting in a lower CORT response when exposed to the same stressor in adulthood. However, the age difference in basal CORT cannot be explained by order effects as samples were collected prior to restraint. Basal CORT differences reflect differences in basal activity, such as locomotion (Beerling et al., 2011). A higher basal CORT concentration in adolescent compared to adult birds may reflect age-related changes in basal activity, but more work is necessary to test this hypothesis.

Adolescent experiences, such as stressors, have sustained effects on adult neophobic behaviors in rodents (Hollis et al., 2013). However, little work has investigated how adult context interacts with developmental experience. Our results are the first to show that stress during early adolescence in zebra finches can influence later-life responses to novelty in a context-dependent manner. In adulthood, familiar conspecifics appear to attenuate or buffer the long-term effects of adolescent stress. Social buffering is a process well documented in social mammals (Hennessey et al., 2009). Social birds, such as affiliative zebra finches, have also shown to be capable of social buffering within adulthood (Coleman and Mellgren, 1994). Our research expands on earlier work to indicate that, in zebra finches, familiar conspecifics can buffer the long-term effects of developmental stress. Zebra finches are highly gregarious birds, typically found in large flocks (Zann, 1996). Social buffering may therefore be one of the benefits of such sociability, but remains to be tested in larger social contexts and across species varying in sociability.

## Appendix A Supplementary data

Supplementary material Image 1
