# Supplementary material for: Long-term effects of adolescent stress on neophobic behaviors in zebra finches are modulated by social context when in adulthood
Source: Horm Behav. 2017 Apr;90:48–55. doi: 10.1016/j.yhbeh.2017.02.004 (PMC5415300; doi:10.1016/j.yhbeh.2017.02.004)
Supplement: Supplementary file 1 — Supplementary material [file mmc1.docx]

**Supplementary Data**

*Supplementary pilot study*

A linear mixed model was used to analyse the pilot study data investigating the corticosterone response to acute restraint in adolescent zebra finches. The model included group, sex, and sampling time as fixed factors (main effects and interactions) with sample time also entered as a repeated measure. The residuals from the analysis had a positive skew (Shapiro-Wilk, p < 0.05), so data were square root transformed prior to the final analysis.

*
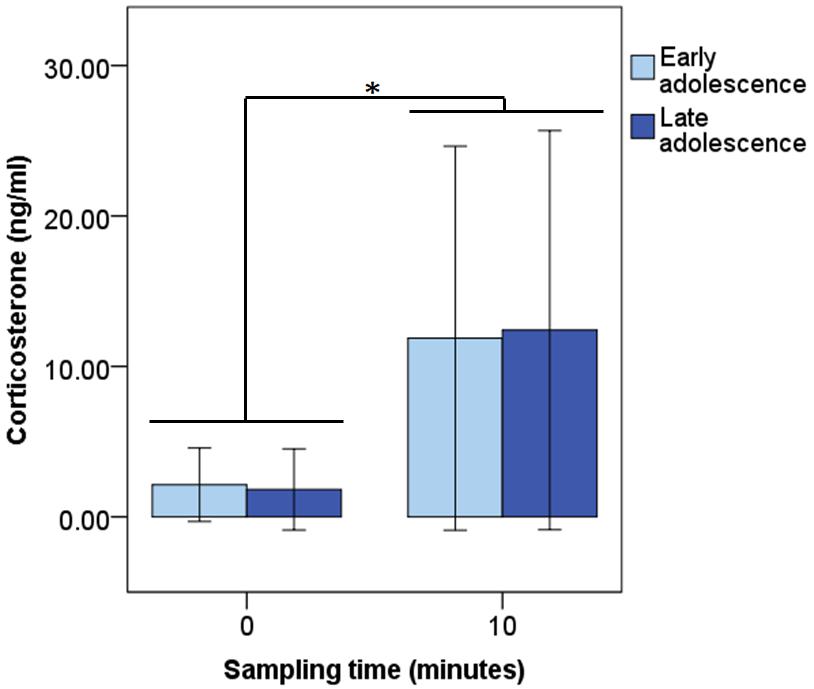
*Birds responded to restraint by secreting more corticosterone, with the concentration of corticosterone higher at 10 mins than baseline (F_1,16_ = 66.588, p < 0.001; Figure S1). The sampling time effect was not affected by age (F_1,16_ = 0.052, p = 0.822), sex (F_1,16_ = 0.022, p = 0.883), or an interaction between age and sex (F_1,16_ = 1.002, p = 0.332). Corticosterone concentration was also not affected by a main effect of age (F_1,16_ = 0.006, p = 0.939), sex (F_1,16_ = 0.067, p = 0.799), or an interaction between age and sex (F_1,16_ = 0.640, p = 0.435).

Figure S1. Corticosterone concentration (ng/ml) in response to restraint in adolescent zebra finches. Birds secreted more corticosterone after 10 mins of restraint compared to baseline regardless of age or sex (p < 0.05 indicated by *). Values are mean + two standard deviations.

*Supplementary results*

| Variable | df | F | p |
| --- | --- | --- | --- |
| sex | 1,58 | 0.069 | 0.794 |
| group | **3,58** | **7.37** | **<0.001** |
| trial | **4,58** | **10.859** | **<0.001** |
| sex*group | 3,58 | 1.881 | 0.143 |
| sex*trial | 4,58 | 1.039 | 0.395 |
| group*trial | **12,58** | **16.086** | **<0.001** |
| sex*group*trial | 12,58 | 0.897 | 0.555 |

Output tables of linear mixed models outlining main effects and interactions are presented in Tables S1-S6. Correlation matrixes for behaviour and hormone associations for each context are presented in Tables S7 and S8. In Table S1, trial refers to five dosing day trials (1-4 vs. 5-8 vs. 9-12 vs. 13-16 vs. 17-20). In Table S4 and Table S5 session refers to pre-exposure vs. exposure to novel compartment comparisons. In Table 6 age refers to adolescent vs. adult corticosterone response comparisons and time refers to blood sampling times (0min vs. 10min vs. 30min). Bold rows indicate significant effects that are discussed in the main manuscript. The legend below each graph reports the proportion of variance explained by the random effects brood size and cage. Cage refers to the home cage of the birds during the group context task.

Table S1. Adolescent latency to enter the dosing box model output. The random effect of brood size explained 9.25% of the variance.

| Variable | Individual Context | | | Group Context | | |
| --- | --- | --- | --- | --- | --- | --- |
|  | df | F | p | df | F | p |
| sex | 1,58 | 0.801 | 0.374 | 1,14.219 | 2.453 | 0.139 |
| group | **3,58** | **7.659** | **<0.001** | 3,14.302 | 0.026 | 0.994 |
| sex*group | 3,58 | 1.159 | 0.333 | 3,14.166 | 0.865 | 0.482 |

Table S2. Latency to enter the novel compartment model output. The random effect brood size explained 4.31% and 2.16% of variance in individual and group contexts, respectively. Cage explained 3.31% of the variance in the group context.

| Variable | Individual Context | | | Group Context | | |
| --- | --- | --- | --- | --- | --- | --- |
|  | df | F | p | df | F | P |
| sex | 1,57.964 | 2.155 | 0.148 | 1,14.105 | 1.51 | 0.239 |
| group | **3,56.657** | **13.488** | **<0.001** | 3,14.186 | 0.203 | 0.893 |
| sex*group | 3,57.968 | 1.201 | 0.318 | 3,14.032 | 0.64 | 0.602 |

Table S3. Duration of time perching in novel compartment model output. The random effect brood size explained 2.06% and 3.31% of variance in the individual and group contexts, respectively. Cage explained 4.72% of the variation in the group context.

| Variable | Individual Context | | | Group Context | | |
| --- | --- | --- | --- | --- | --- | --- |
|  | df | F | p | df | F | p |
| sex | 1,58 | 1.025 | 0.316 | 1,13.749 | 0.165 | 0.691 |
| group | 3,58 | 1.756 | 0.166 | 3,13.734 | 0.12 | 0.947 |
| session | **1,58** | **128.52** | **<0.001** | **1,58** | **107.933** | **<0.001** |
| sex*group | 3,58 | 2.42 | 0.075 | 3,13.697 | 0.525 | 0.673 |
| sex*session | 1,58 | 1.597 | 0.211 | 1,58 | 0.589 | 0.446 |
| group*session | 3,58 | 6.252 | 0.001 | 3,58 | 0.402 | 0.752 |
| sex*group*session | 3,58 | 4.206 | 0.009 | 3,58 | 0.468 | 0.706 |

Table S4. Number of head turns during novel environment test model output. The random effect of brood size explained 2.43% and 0.82% of variance in individual and group contexts, respectively. Cage explained 1.75% of the variance in the group context.

| Variable | Individual Context | | | Group Context | | |
| --- | --- | --- | --- | --- | --- | --- |
|  | df | F | p | df | F | p |
| sex | 1,58.114 | 0.002 | 0.967 | 1,15.403 | 0.04 | 0.844 |
| group | 3,59.147 | 0.576 | 0.633 | 3,18.793 | 1.036 | 0.399 |
| session | **1,58.120** | **20.256** | **<0.001** | **1,58.742** | **70.461** | **<0.001** |
| sex*group | 3,59.189 | 0.147 | 0.931 | 3,18.793 | 0.145 | 0.932 |
| sex*session | 1,58.119 | 0.165 | 0.686 | 1,58.742 | 1.023 | 0.316 |
| group*session | 3,58.408 | 0.197 | 0.898 | 3,59.136 | 0.033 | 0.992 |
| sex*group*session | 3,58.405 | 0.551 | 0.649 | 3,59.136 | 1.603 | 0.198 |

Table S5. Number of perch hops during novel environment test model output. Random effect of brood size explained 4.31% and 2.16% of variance in individual and group contexts, respectively. Cage explained 7.24% of the variance in the group context.

| Variable | Df | F | p |
| --- | --- | --- | --- |
| sex | 1,55.331 | 0.286 | 0.595 |
| group | 3,55.9 | 0.887 | 0.454 |
| time | **2,58.034** | **220.357** | **<0.001** |
| age | **1,57.942** | **16.378** | **<0.001** |
| sex*group | 3,56.282 | 2.237 | 0.094 |
| sex*time | 2,58.034 | 1.065 | 0.351 |
| sex*age | 1,57.942 | 0.086 | 0.771 |
| group*time | 6,58.030 | 0.398 | 0.877 |
| group*age | 3,57.938 | 0.536 | 0.66 |
| time*age | **2,57.934** | **20.178** | **<0.001** |
| sex*group*time | 6,58.030 | 0.502 | 0.804 |
| sex*group*age | 3,57.938 | 1.238 | 0.304 |
| sex*time*age | 2,57.934 | 2.078 | 0.134 |
| group*time*age | 6,57.928 | 0.603 | 0.727 |
| sex*group*time*age | 6,57.928 | 0.377 | 0.890 |

Table S6. Corticosterone response to a capture-restraint stressor model output. The random effect of brood size explained 8.14% of variance.

Table S7. Correlations between individual context novel environment task variables and corticosterone concentrations in response to capture and restraint during adolescence and adulthood. Significant correlations (p < 0.05) are in bold.

Table S8. Correlations between group context novel environment task variables and corticosterone concentrations in response to capture and restraint during adolescence and adulthood. Significant correlations (p < 0.05) are in bold.
